# Editorial: Population aging and older health in an era of digitalization: empirical findings and implications

**DOI:** 10.3389/fpubh.2024.1414013

**Published:** 2024-05-16

**Authors:** Wei Guo, Xiangnan Chai, Zhenmei Zhang, Zhihao Ma

**Affiliations:** ^1^Department of Social Work and Social Policy, School of Social and Behavioral Sciences, Nanjing University, Nanjing, China; ^2^Department of Sociology, School of Social and Behavioral Sciences, Nanjing University, Nanjing, China; ^3^Department of Sociology, College of Social Science, Michigan State University, East Lansing, MI, United States; ^4^Computational Communication Collaboratory, School of Jouralism and Communication, Nanjing, China

**Keywords:** population aging, older adults' health, digitalization, empirical findings, health implications, international perspective, editorial summary

In this Research Topic, our objective is to solicit research that centers on the health of older adults in the context of the digital era. The phenomenon of population aging has emerged as a significant concern across various regions, including middle-income countries like China (1), as well as in most high-income countries such as Korea (2), Japan (3), the U.S. (4), Canada (5), and many European nations (3). Furthermore, the rapid global advancement of digital technology has profoundly impacted health management and enhancement, influencing healthcare delivery models (6) and timely health communications in users' daily lives (7). Within this context, the examination of how digital technologies shape older adults' health has emerged as a central Research Topic in gerontological and sociological studies.

We received a considerable volume of submissions, and following meticulous editorial and peer review procedures, we ultimately disseminated nine articles of high quality. Among these contributions, eight specifically address the health of older adults in China, while one centers on older adults' health in Korea. All the articles closely align with the overarching theme of older adults' health in the digital era. Notably, each study adopts a quantitative approach. While some investigations rely on nationally representative data, others draw from community-based survey data with relatively modest sample sizes.

The research objectives and outcomes of the nine articles can be summarized as follows.

The study conducted by Wang et al. delved into the advantageous effects of internet usage on support for older adults. Their research findings suggest that increased frequency of internet use is associated with higher probabilities of older Chinese adults engaging in activities such as purchasing commercial pension insurance, accepting formal care, and fostering independence in later life. These positive outcomes are attributed to the dissemination of information and the maintenance of social connections facilitated by internet access.

Xiao et al. explored the relationship between social security enhancements and the health outcomes of older adults actively participating in digital financial inclusion systems. Their results demonstrate that strengthening social security has a positive impact on the wellbeing of older adults. Notably, this influence is more pronounced among older Chinese men than women. Additionally, rural residents derive significant benefits from improved social security provisions, leading to the most favorable health outcomes.

Nan et al. conducted a study to examine the relationship between internet usage and depression among older Chinese adults. Their findings revealed that specific types of internet use are associated with varying levels of depression. For instance, engaging in activities such as chatting via WeChat, browsing online videos, and shopping online is positively correlated with lower levels of depression. These internet activities also contribute to improved interpersonal relationships, which may subsequently reduce depression in older Chinese adults. Conversely, other online activities, such as playing games or online learning, do not show a significant association with depression levels in this population.

Ren et al.s' research focuses on formalizing the domestic service industry to enhance the provision of care services for older adults. Through model construction and simulation analysis, they identified effective strategies. These include increasing the market share of domestic enterprises with employee management systems, implementing subsidy programs for clients, and establishing evaluation and supervision mechanisms. These measures contribute to the overall support and quality of care services.

In their study, Li et al. investigated the factors influencing individuals' intention to use health management systems, which subsequently impact their actual usage. The key factors identified for older adults' behavioral intention to accept a health management system include their expectations of effort, social influences, perceived value, performance expectations, perceived interactivity, and perceived risk.

Wei and Guo conducted a study examining the impact of smart device usage on the self-reported physical and psychological health of older Chinese adults. Their findings indicate that the use of smart devices has a positive effect, particularly among older adults residing in urban areas or those of advanced age. Furthermore, the personal attitudes of older Chinese residents toward smart devices partially mediate the relationship between smart device usage and health outcomes.

Park et al. investigated the relationship between the accessibility of information and communication technology (ICT) and the psychological wellbeing of older Koreans. The COVID-19 pandemic intensified reliance on ICT, shifting engagement from self-motivated to socially passive modes. Despite an initial positive association between ICT use and mental health among older adults in Korea, this link weakened during the pandemic. Notably, heterogeneity exists based on age, sex, and rural residence, with older females in their 70's experiencing the most pronounced effects.

Fan et al. explored the potential of digital health technologies to enhance frailty detection and diagnosis efficiency among older adults. Their investigation revealed that a composite model, which integrated comprehensive geriatric assessment (CGA) and gait parameters, effectively predicted frailty. Remarkably, this composite model outperformed individual features. After feature selection, machine learning models demonstrated significant improvement, with performance gains ranging from 4.3 to 11.4%. Key predictors included large-step walking speed, average step size, age, total step walking distance, and Mini Mental State Examination score.

Liu et al. conducted a study to investigate the influence of digital health literacy on the health-related quality of life (HRQoL) of community-dwelling older Chinese adults. Their findings reveal a positive association between digital health literacy and HRQoL. Importantly, health-promoting lifestyle serves as a mediator in this relationship, emphasizing the need for relevant management institutions, communities, and families to prioritize the cultivation of digital health literacy among older adults.

In summary, the collective evidence from the nine articles featured in our Research Topic unequivocally demonstrates that the advancement of digital and information technology significantly and predominantly benefits the physical, psychological, and social wellbeing of older adults. These findings underscore the necessity of enhancing older adults' access to digital technologies and emphasize the importance of enhancing their digital literacy, particularly in the context of healthcare-related technologies.

## Author contributions

WG: Investigation, Resources, Supervision, Writing – original draft, Writing – review & editing. XC: Resources, Supervision, Writing – original draft, Writing – review & editing. ZZ: Investigation, Resources, Supervision, Writing – review & editing. ZM: Resources, Writing – review & editing.
